# Spin coating on a budget: A 3D-Printed all-mechanical alternative for cost-effective thin-film deposition

**DOI:** 10.1016/j.ohx.2024.e00547

**Published:** 2024-06-25

**Authors:** Apostolos Kalafatis, Lazaros Theofylaktos, Thomas Stergiopoulos

**Affiliations:** a Institute of Nanoscience and Nanotechnology, National Center for Scientific Research “Demokritos”, Aghia Paraskevi, Athens 15341, Greece; b Department of Chemistry, Aristotle University of Thessaloniki, 54124 Thessaloniki, Greece

**Keywords:** *Spin coating*, 3D-printing, Open-source lab tools, Thin-film deposition, Solar cells

## Abstract

Spin coating stands out as the most employed thin-film deposition technique across a variety of scientific fields. Particularly in the past two decades, spin coaters have become increasingly popular due to the emergence of solution-processed semiconductors such as quantum dots and perovskites. However, acquiring commercial spin coaters from reputable suppliers remains a significant financial burden for many laboratories, particularly for smaller research or educational facilities. Prompted by the simple mechanical principles of the device, in this work, we present a 3D-printed analogue that can be printed and assembled in under 10 h and costs less than 5 euros per device. The operating principle is fully mechanical since the rotating motion is induced by gas flow. It does not require any additional components such as DC motors, motor drivers, circuitry or software and thus it can be fully operational off the grid. Additionally, the gas flow generates a purging effect that was found to be rather advantageous for film formation. To prove the effectiveness of this device, we have employed it to fabricate planar thin-film antimony sulfide (Sb${}_{2}$S${}_{3}$) solar cells. The optoelectronic characteristics of solar cells revealed noteworthy improvements, particularly in terms of repeatability, when compared to those fabricated with a commercial spin coater.

## Graphical abstract

**Figure:**
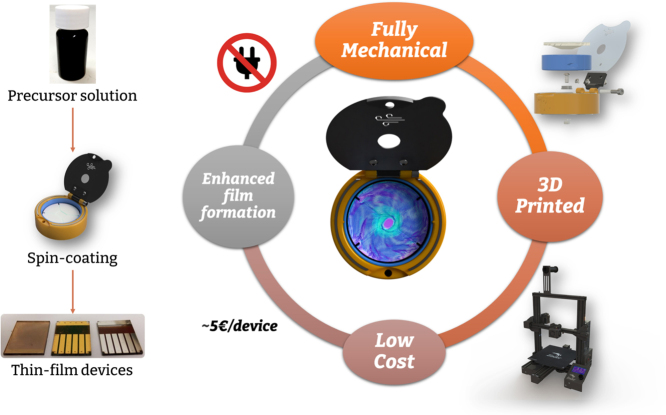

**Specifications table**

**Table:** 

**Hardware name**	*Turbo-Coat*

**Subject area**	• *Engineering and material science* • *Chemistry and biochemistry* • *Thin films* • *Photovoltaics* • *General*

**Hardware type**	• *Thin-film deposition*

**Closest commercial analog**	Commercial Spin Coaters

**Open source license**	Creative Commons Attribution-ShareAlike 4.0 International (CC BY-SA 4.0)

**Cost of hardware**	5€

**Source file repository**	https://doi.org/10.5281/zenodo.10521930

## 1 Hardware in context

Spin coaters are electromechanical devices that are widely used in thin-film deposition methods, playing a crucial role in various scientific and industrial applications. They are renowned for their simplicity, efficiency, and ability to produce uniform thin films on various substrates. In addition, spin coating is almost exclusively used in solution-processed optoelectronic devices (solar cells, sensors, LEDs etc.) on a laboratory scale.

A spin coating event commonly involves a solution or dispersion of a specific material to be applied onto a substrate. The substrate is fixed onto a holder (also referred to as a chuck) which rotates with a specific frequency. The centrifugal force induced by this rotation causes the liquid to spread uniformly across the surface of the substrate, thus creating a thin, homogeneous film. While spinning, the film thickness reduces until disjoining pressure effects cause the film to reach an equilibrium thickness or turn into a solid due to a dramatic rise in viscosity due to solvent evaporation. The final result is influenced by centrifugal force, viscous forces, and solvent evaporation [1]. This process facilitates the deposition of thin films with controlled thickness, smooth surfaces, and minimal defects. Common solutions/dispersions include photoresists used in photolithography, quantum dot dispersions, precursor solutions for semiconducting films and oxide precursors for microelectronics. Typical substrates could be glass (e.g., microscopy slides), glass coated with transparent conductive oxides (e.g., fluorine-doped tin oxide), plastic and microchips. The cost for a commercial lab-scale spin coater ranges from 500 euros to a couple of thousands depending on the features and substrate requirements.

Due to the recent rise of 3D printing, low-cost 3D-printed lab equipment is quickly developing in various scientific fields including microfluidics [2], [3], optics [4], chromatography [5] and general hardware [6], [7], [8]. That said, some attempts have been made to recreate 3D-printed spin coaters with significantly lower cost per device. Most open-source designs use computer hard-drive parts that are adapted to fit a chuck [9], [10]. In a recent study, Rubio et al. [11] proposed a spin coater design using 3D printing and brushless DC motors commonly found in RC vehicles. Although previous spin coater designs significantly reduced costs, they all relied on commercial electromechanical spin coater concepts.

**Fig. 1:**
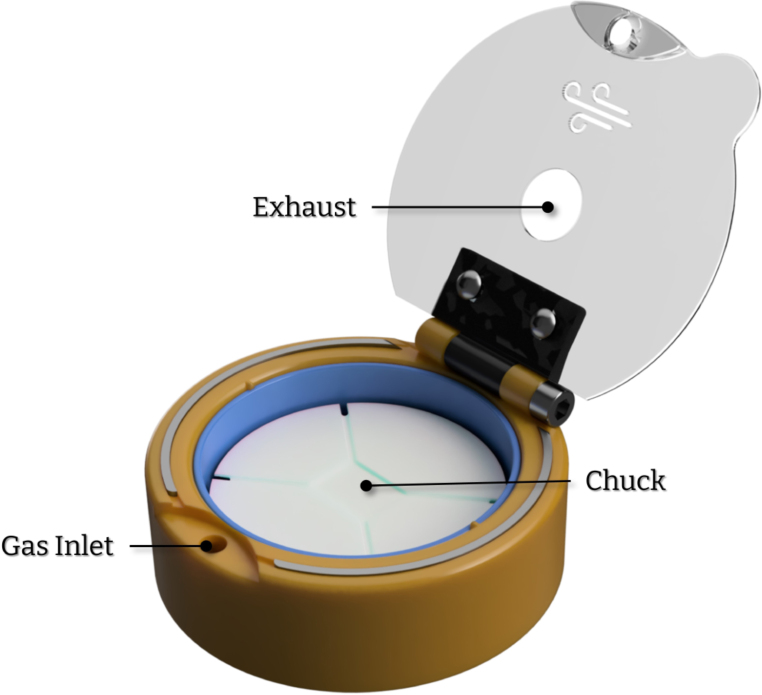
CAD render of Turbo-Coat.

Here, we present a 3D-printed, fully mechanical, cost-effective, adaptable, and open-source alternative capable of producing high-quality thin films. The device (Fig. 1) can be printed and assembled in less than 10 h without requiring expensive tooling, hardware or software. Consequently, the total cost of materials and hardware is less than 5 euros per unit. Additionally, the device possesses a unique feature that is found to be advantageous for semiconducting thin films. With this work, we strive to provide affordable alternatives for research and educational institutions, while also advancing the field of open-source lab equipment that is freely available for all to use and modify.

**Fig. 2:**
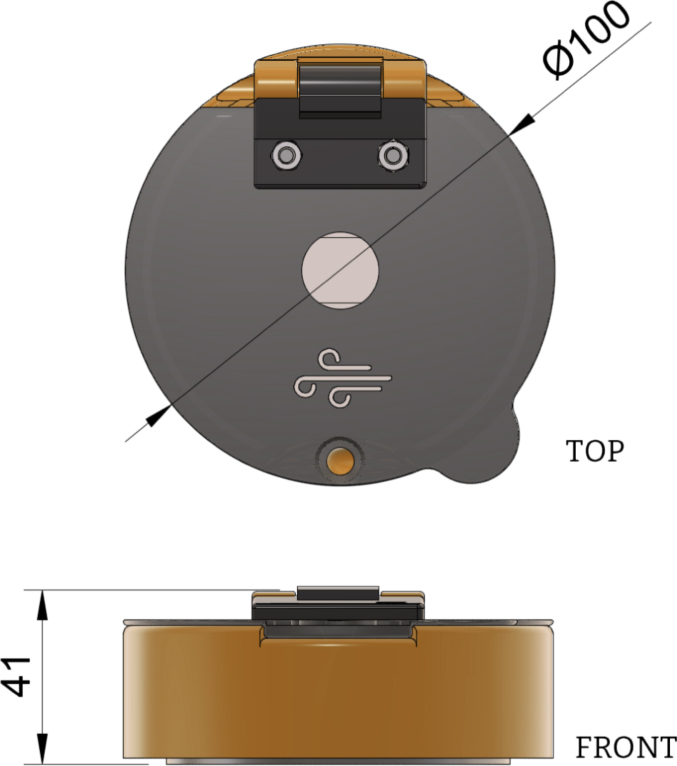
Dimensions of Turbo-Coat (mm).

## 2 Hardware description

The device (Turbo-Coat) is operated using regular compressed air or compressed inert gases (e.g., N${}_{2}$, Ar etc.) commonly available in research labs. This feature eliminates the need for electric motors, motor drivers, electronic circuits, microcontrollers and software interfaces. Additionally, the dimensions (Fig. 2), weight (ca. 180 g) and wireless design of the device are perfect for on-site and off-grid operation. The design is optimized to be easily 3D-printed using commercial low-cost fused deposition modelling (FDM) or stereolithography (SLA) 3D printers (e.g., Ender 3, Voron, Anycubic Photon Mono X.). In the case of the FDM-printed device, to further reduce the cost and improve sustainability, more than 90% by weight can be printed using recycled polylactic acid (PLA) and polyethylene terephthalate (PET) filaments. The remaining 10% of the device’s weight is printed with polypropylene (PP) and thermoplastic elastomer (TPE) filaments. The choice of filaments is made depending on the relative function of the resulting parts. The parts of the spin coater that do not come into contact with chemicals or are not subjected to heavy strain can be safely printed with PLA filament. For the parts in need of maintaining chemical resistance PET and PP filaments are chosen. Of course, the choice of filaments can be changed to suit any specific needs (i.e., from all-PP-printed designs to PLA/TPU designs). Finally, the assembly only requires inexpensive hardware readily found in hardware stores (bolts, nuts, washers and bearings).

### 2.1 Fully-mechanical operation

**Fig. 3:**
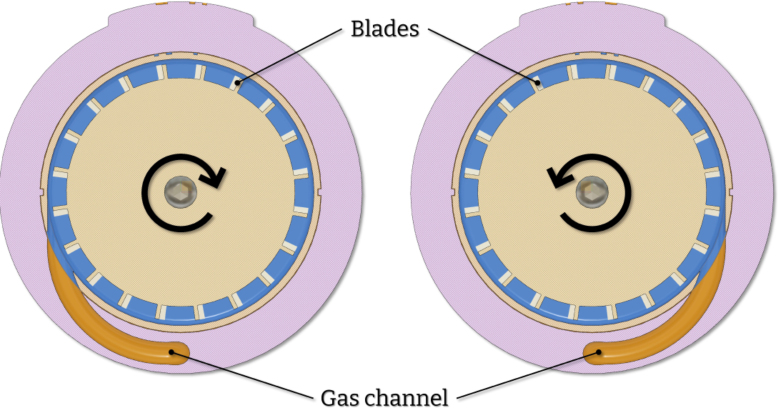
XY-section.

**Fig. 4:**
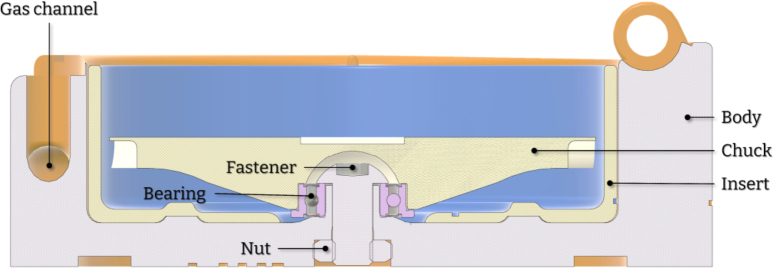
YZ-section.

**Fig. 5:**
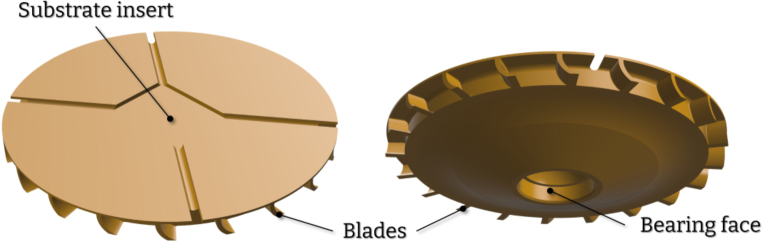
CAD rendering of the chuck.

To achieve fully-mechanical operation, the device utilizes a gas flow directed towards a specially designed chuck (Fig. 5). The body of the device incorporates an internal hollow channel that guides the gas flow. When pressure is applied to the inlet, gas is transferred through this channel and exits the body in a direction tangent to the chuck (Fig. 3). As the gas flow intercepts the blades on the chuck, it induces the required rotation. The result is the angular acceleration of the chuck with no need for an electric motor. The frequency of rotation is defined by regulating the applied pressure at the inlet. The design consists of seven major parts, a section analysis in the YZ plane highlighting the operating principle is shown in Fig. 4. The body of the device serves as the deposition chamber and forms the base for mounting all other components. The insert ensures the required gas velocity by providing correct tolerances and is easily removable and replaceable. Finally, the rotating chuck is press-fitted onto a flanged bearing which itself is secured to the body with a fastener and a nut.

### 2.2 Design adaptability

**Fig. 6:**
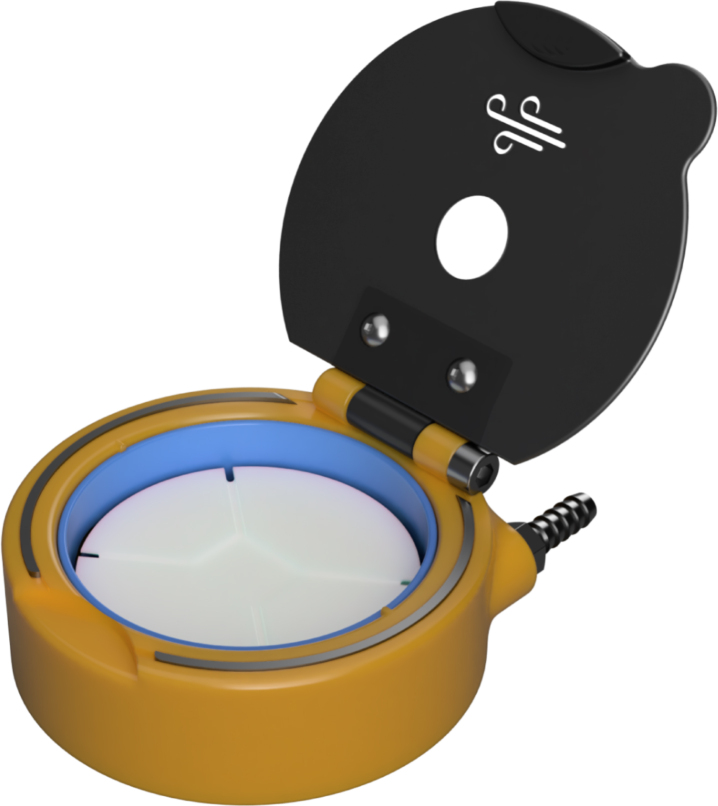
CAD rendering of the device equipped with a gas fitting.

CAD files are easily adaptable to accommodate diverse needs. For instance, the chuck can be redesigned to accommodate various substrates, and the body can be modified to allow connection to a gas fitting (Fig. 6). In the latter scenario, the device can be securely fixed in place, and airflow can be controlled using a flow valve. Here, we will introduce the simplest version, utilizing an air blow gun to achieve the necessary airflow. Additionally, two different versions of the 3D-printed parts are provided to achieve different rotational directions. Non-symmetrical parts are mirrored to achieve counterclockwise rotation (Fig. 3). Design files for the clockwise and counterclockwise rotation are designated as PART_R and PART_L respectively. The as-presented material selection was found to work well for various organic solvents and aqueous solutions. However, material alterations can be made depending on the required chemical resistance of each part. In any case, we recommend printing the chuck with flexible filaments (TPU 95 A or PP) because this part is press-fitted onto the bearing.

### 2.3 Gas flow effects on film formation

The physics governing thin film formation by spin coating is well established. Initially, Emslie et al. [12] described this process as a one-dimensional system involving a Newtonian fluid on an infinite rotating plate. However, this early model only accounted for centrifugal forces, neglecting evaporation effects. Later, Meyerhofer [13] improved upon the previous model including constant evaporation rates and showed that spin coating involves two stages; The first stage lasts for a few milliseconds and is characterized by the thinning of the film primarily due to the radial convection outflow. In the second stage, solvent mass transfer becomes the dominant process and is controlled by the diffusion of the solvent in the film and the solvent’s partial pressure [14]. In the same work, Meyerhofer emphasizes that the rate of evaporation greatly depends on how quickly the vapour phase above the liquid is replaced with unsaturated continuous gas. It is expected that the evaporation rate would be heavily influenced by the flow of this vapor phase and therefore by the rotational speed of the chuck. However, the gas flow induced by the rotating chuck generates spiral vortices (Ekman spirals), disrupting material transport to and from the substrate, resulting in non-uniform thickness distribution of the films in both spin coating and chemical vapour deposition techniques [15].

**Fig. 7:**
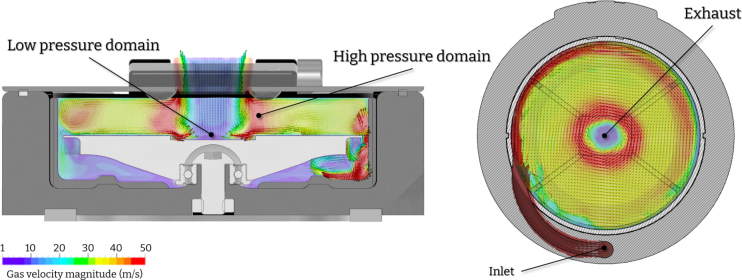
Static CFD simulation [17] of the device during operation. The pressure applied at the inlet is 3 bar and the exhaust port is set to atmospheric pressure (1 atm). (For interpretation of the references to colour in this figure legend, the reader is referred to the web version of this article.)

To produce semiconducting thin films, controlling factors like temperature, humidity, and atmospheric composition are crucial. This can be achieved by performing the spin coating process in gloveboxes filled with nitrogen or argon gas to create inert gas conditions. Moreover, it is important to maintain continuous inert gas flow in the glovebox during each spin coating event [16]. Despite these measures, thickness inconsistencies may persist due to Ekman spirals, affecting thin-film device properties. Thickness variations across the film can affect the crystallinity, defect density, and proper formation of ohmic contacts in optoelectronic thin-film devices. To address this issue, our design ensures a continuous gas flow inside the deposition chamber during the spin coating process. Pressurized gas used for chuck rotation also purges the chamber. Computer flow dynamics (CFD) analysis (Fig. 7) reveals two distinct pressure domains that generate a single vortex in the proximity of the sample. The geometry of the exhaust port ensures that the generated vortex is of sufficient size to have a uniform effect across the sample. This feature effectively eliminates any inconsistencies that may arise from Ekman spirals localized on different domains of the sample, ensuring uniform thickness across the surface.

Moreover, a high-pressure domain is generated along the circumscribed circle that shapes the exhaust port (shown in red in Fig. 7) while a low-pressure domain is formed at the centre of the same circle (shown in blue in Fig. 7). This creates a pressure drop on the surface of the sample, which coincides with the vapour pressure required for solvent molecules to transition from the liquid to the gaseous phase. The reduced pressure leads to fewer collisions between gas molecules and the surface of the liquid, enabling more efficient phase-change of individual molecules. The presence of this vortex due to the low/high-pressure domains has a synergetic effect on the efficiency of evaporation. The evaporated solvents are rapidly removed and replaced with new unsaturated gas so that the champer conditions always favour the evaporation of the solvent.

## 3 Design files summary

**Table:** 

Design filename	Designator	File type	Open source license	Location of the file
BODY_L.stl	PN_1	.stl	CC BY-SA 4.0	10.5281/zenodo.10521930
BODY_R.stl	PN_1	.stl	CC BY-SA 4.0	10.5281/zenodo.10521930
INSERT_L.stl	PN_2	.stl	CC BY-SA 4.0	10.5281/zenodo.10521930
INSERT_R.stl	PN_2	.stl	CC BY-SA 4.0	10.5281/zenodo.10521930
LID.stl	PN_3	.stl	CC BY-SA 4.0	10.5281/zenodo.10521930
HINGE.stl	PN_4	.stl	CC BY-SA 4.0	10.5281/zenodo.10521930
CHUCK_L.stl	PN_5	.stl	CC BY-SA 4.0	10.5281/zenodo.10521930
CHUCK_R.stl	PN_5	.stl	CC BY-SA 4.0	10.5281/zenodo.10521930
RINGS.stl	PN_5	.stl	CC BY-SA 4.0	10.5281/zenodo.10521930
ASSEMPLY_L.step	PN_1-5, PN_H1-6	.step	CC BY-SA 4.0	10.5281/zenodo.10521930
ASSEMPLY_R.step	PN_1-5, PN_H1-6	.step	CC BY-SA 4.0	10.5281/zenodo.10521930
ALL_L.3mf	PN_1-5	.3mf	CC BY-SA 4.0	10.5281/zenodo.10521930
ALL_R.3mf	PN_1-5	.3mf	CC BY-SA 4.0	10.5281/zenodo.10521930

**FILE_X.stl** files contain the individual parts of the device. For non-symmetric parts, two files are provided; FILE_L.stl and FILE_R.stl for counterclockwise and clockwise rotation respectively. The corresponding .step files for individual parts are also available in the repository.

**ASSEMPLY_X.step** files are editable containing 3D-printed parts and hardware for both rotational directions.

**ALL_X.3mf** files contain all the 3D-printable parts for both rotational directions.

## 4 Bill of materials summary

**Table:** 

Designator	Component	Number	Cost per unit - €	Total cost - €	Source of materials	Material type
**3D prints - cost is based on**Table 1						

PN_1	Body	1	1.1	1.1	Polymaker	Polymer (PLA)

PN_2	Insert	1	0.46	0.46	Polymaker	Polymer (PET-G)

PN_3	Lid	1	0.23	0.23	Polymaker	Polymer (PET-G)

PN_4	Hinge	1	0.1	0.1	Polymaker	Polymer (PET-G)

PN_5	Chuck	1	1	1	Verbatim	Polymer (PP)

PN_6	Antislip/seal rings	1	0.19	0.19	Esun, Esun	Polymer (TPU/TPE)

**Hardware**						

PN_H1	Fastener M6x12	1	0.1	0.1	Aliexpress	Metal

PN_H2	Bearing F688zz	1	0.6	0.6	Aliexpress	Metal

PN_H3	Nut M4	2	0.05	0.1	Aliexpress	Metal

PN_H4	Fastener M4x5	2	0.05	0.1	Aliexpress	Metal

PN_H5	Fastener M6x40	1	0.1	0.1	Aliexpress	Metal

PN_H6	Nut M6	1	0.05	0.05	Aliexpress	Metal

## 5 Build instructions

### 5.1 FDM 3D printing

**Fig. 8:**
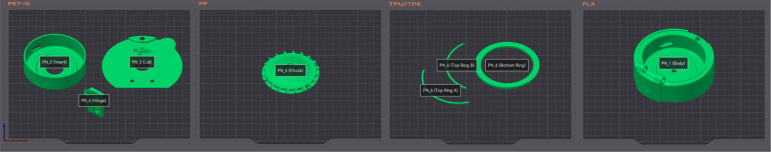
Suggested orientations for FDM 3D printed parts.

**Fig. 9:**
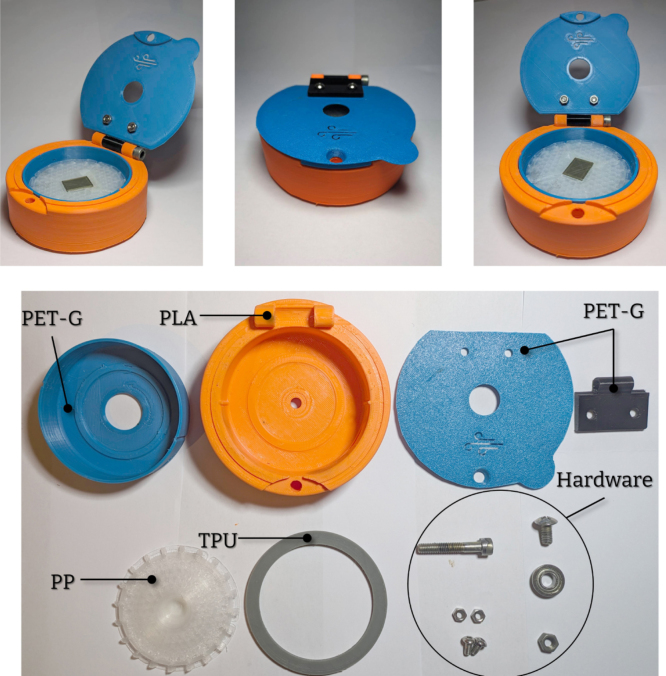
As-printed parts and hardware.

The 3D-printed parts (PN_1-PN_6) are printed with an inexpensive and commercially available 3D printer (Ender3 Neo) using a 0.4 mm nozzle and 1.75 mm filaments. These parts are easily printable using standard approaches for PLA, PET-G, PP, and TPU/TPE filaments. While a direct drive extruder facilitates printing with flexible filaments, other configurations can be used with proper calibration. Special attention should be paid when printing the chuck (PN_5) due to the nature of the filament (PP). As the only rotating 3D-printed part, it must be dimensionally accurate and well-balanced. Imperfections could result in vibrations, affecting device performance. Since PP filament does not adhere reliably to typical build plates but does adhere to PP surfaces, we recommend covering the printer’s build plate with PP tape (applying two layers of standard masking tape should be sufficient). Because of the strong layer adhesion of PP prints, conventional support structures are not very effective. Instead, we recommend pausing the print before the bridging layer and inserting a glass substrate of the same dimensions as the substrate insert. PP does not adhere to glass, allowing easy removal of the glass substrate afterwards. This ensures a flat and dimensionally accurate surface for the substrates. As mentioned in Section 2.2, the chuck could also be printed with other flexible filaments that are easier to print (e.g., TPU or TPE). The suggested print orientations and settings for 3D-printed parts are shown in Fig. 8 and Table 1 respectively. The as-printed parts are shown in Fig. 9.

**Table 1:** Proposed printing settings and filament usage for FDM 3D-printing.

	Filament	Layer height	Walls	%Infill	Supports	Used filament (m)	Time (h)	
Body	PLA	0.2	3	20	Yes	17.6	3.1	
Instert	PET-G	0.2	3	100	Yes	6.5	1.2	
Lid	PET-G	0.2	2	100	No	3.7	0.7	
Hinge	PET-G	0.2	4	100	Yes	1.6	0.6	
Antislip/seal rings	TPU	0.2	3	100	No	2.1	0.7	
Chuck	PP	0.2	4	40	Custom	4.7	2.1	

							8.4	Total

### 5.2 SLA 3D printing

The device can also be printed using SLA 3D printers. This method guarantees greater dimensional precision and eliminates potential issues associated with the FDM method. Especially details like the air channel and the seat of the bearing will particularly benefit from the increased resolution of SLA printing. It is advised to use high-performance resins (engineering resins) that offer good mechanical performance and good chemical resistance. Similar to the FDM method, the chuck must be printed with a flexible and chemically resistant resin, depending on the intended application.

### 5.3 Assembly

**Fig. 10:**
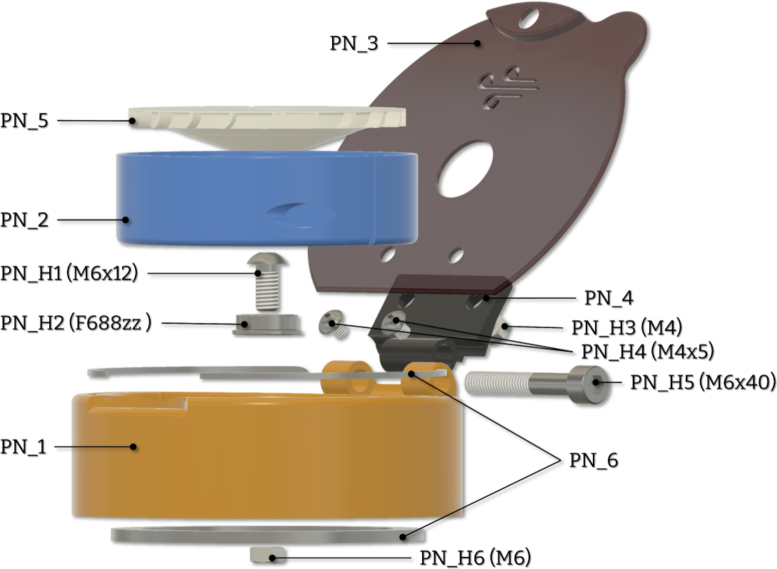
Exploded view of the device.

The device is assembled based on the exploded diagram in Fig. 10

• Attach the antislip rings (PN_6) to the body (PN_1). The tolerances are chosen for a friction fit but general-purpose glue may need to be applied.
• Insert the bolt (PN_H1) and bearing (PN_H2) to the body and tighten the nut (PN_H6). Ensure smooth and frictionless operation of the bearing after tightening.
• Attach the hinge (PN_4) to the body with the appropriate bolt (PN_H5) and tighten. The dimensions of the hole are set so it operates as a self-threaded screw. Ensure smooth operation of the hinge.
• Attach the lid (PN_3) and secure it with the appropriate bolts (PN_H4) and nuts (PN_H3). Ensure a good alignment of the lid and the body.
• Slide the insert (PN_2) in place using the alinement slots. Confirm that the hole for the air channel coincides with the hole on the body.
• Press-fit the chuck (PN_5) on the bearing by applying slight pressure in the centre. Ensure the chuck spins freely and is correctly centred.

**Fig. 11:**
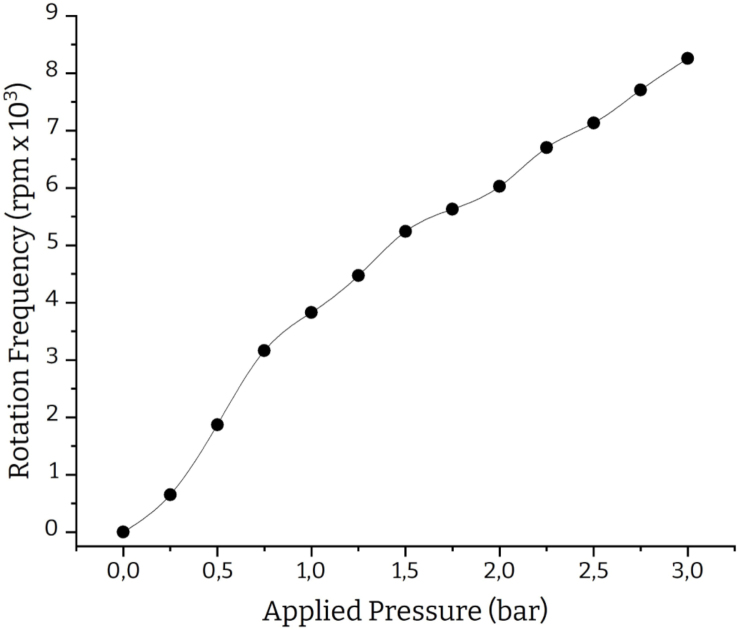
Pressure-rpm calibration curve.

## 6 Operation instructions

To execute a spin coating insert the substrate on the chuck, close the lid and apply the desired solution/dispersion onto the substrate with a pipette. Place the air gun in the corresponding inlet hole on the lid and apply the required pressure. The as-presented device (Fig. 9) with external dimensions shown in Fig. 2 is designed to operate in the 800–8000 rpm. Furthermore, due to the lack of electronics, the spin coating duration needs to be monitored with a separate device. To ensure safe operation, the spin coating must always be performed under a fumehood with adequate updraft or inside a glovebox. It is important to note that there is pressure buildup in the deposition chamber due to the specific nature of the device. This pressure is released through an opening on the lid. The gas stream exiting the lid may contain potentially hazardous chemicals and solvents. Therefore, users should never remain above the device while it is spinning. Finally, due to the 3D-printed nature of the device, it is advised not to exceed spin speeds greater than 8k rpm.

### 6.1 Device calibration

Any method capable of generating a pressure-rpm calibration curve will suffice. The simplest way is to use a handheld LASER tachometer to collect the corresponding rpm values for various applied pressures. The calibration curve used for our experiments is shown in Fig. 11. Another way to calibrate the device could be using an Arduino-based tachometer equipped with an IR or LASER emitter coupled to the corresponding photodetector. We suggest using the LASER tachometer method for its simplicity, performance and off-grid operation. The responsiveness of the 3D-printed devices is based on various factors (e.g., material selection, layer height, number of walls, gas selection, etc.) so a new calibration curve has to be created for every device. Finally, it is recommended to perform the calibration with a loaded chuck to account for the exact mass of the rotating components.

### 6.2 Validation in Sb${}_{2}$S${}_{3}$ thin-film solar cells

**Fig. 12:**
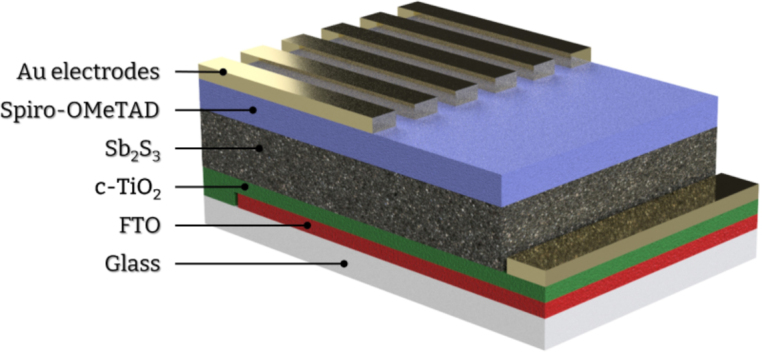
Sb${}_{2}$S${}_{3}$ Solar cell structure.

To assess the performance of the device in thin film deposition we proceeded with the fabrication of Sb${}_{2}$S${}_{3}$ thin film solar cells. These photovoltaic devices consist of semiconducting thin films typically in the range of nanometers (Fig. 12).

A homogeneous film formation is crucial in these applications to eliminate defects and ensure optimal ohmic contacts between heterojunctions. Therefore, we consider it an ideal test to assess the purging effect on film quality. To do that we focused on the Sb${}_{2}$S${}_{3}$ thin film. Following previously reported experimental procedures [18], we fabricated two batches of solar cells; In the first batch (denoted as Turbo-Coat), the Sb${}_{2}$S${}_{3}$ film was deposited using our spin coater while for the second batch (denoted as Reference), we used a commercially available analogue (Ossila spin coater). In both cases, the depositions were carried out in a nitrogen-filled glovebox. Pressurized nitrogen was also used to induce the rotation in our device. The spin coating of the Sb${}_{2}$S${}_{3}$ thin film was executed at 2000 rpm for 30 s in both experiments. The corresponding nitrogen pressure for our device was determined using the presented calibration curve (Fig. 11) and monitored with a LASER tachometer (ca. 0.5 bar).

**Fig. 13:**
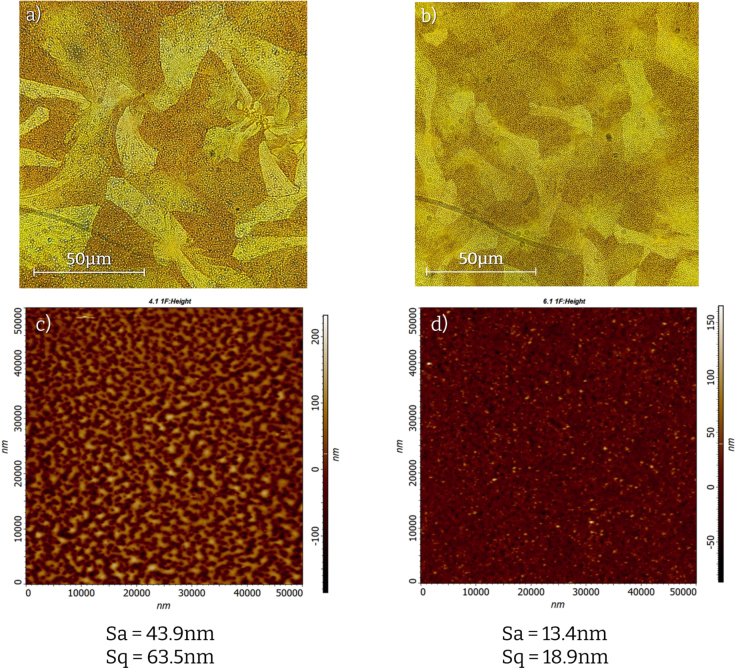
OM and AFM images of Sb${}_{2}$S${}_{3}$ thin films. (a) OM of sample prepared with reference spin coater. (b) OM of sample prepared with Turbo-Coat. (c) AFM of sample prepared with reference spin coater. (d) AFM of sample prepared with Turbo-Coat.

Optical microscopy (OM) and atomic force microscopy (AFM) were employed to evaluate the quality of the resulting thin films (Fig. 13). In OM, the films appear similar but in the case of the reference samples white dots appear throughout the surface. These defects are vastly eliminated in the case of Turbo-coat samples. Similarly, AFM images show a notable decrease in surface roughness for samples prepared with the Turbo-coat spin coater.

**Fig. 14:**
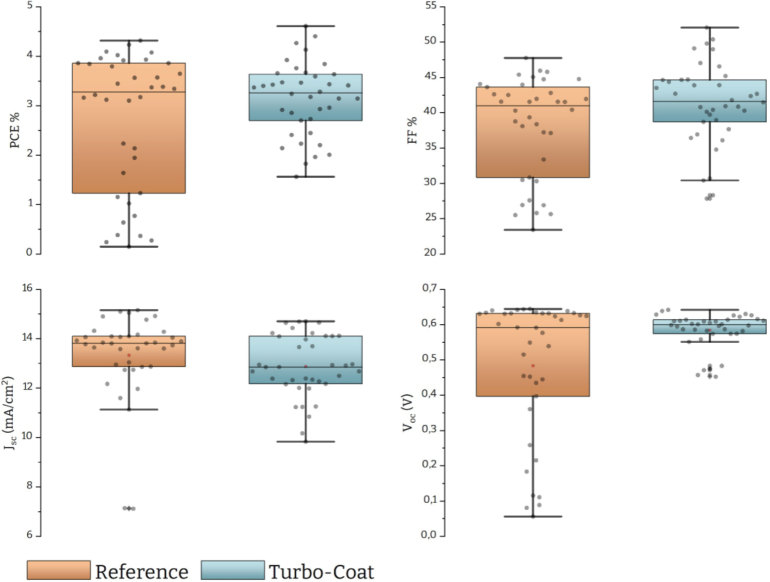
Optoelectronic characteristics of Sb${}_{\text{2}}$S${}_{\text{3}}$ solar cells fabricated with reference and Turbo-Coat spin coaters.

Each solar cell is divided into 4 distinct regions (pixels) and the current–voltage (J-V) curve is measured using a solar simulator for each pixel. We fabricated 5 solar cells per batch, yielding 20 pixels. The number of operational pixels (PCE>0.1%) was 19 for both batches (95% of operational pixels). The statistical analysis was conducted over these 19 operational pixels in forward and reverse bias (38 data points). The optoelectronic characteristics are presented in Fig. 14 and demonstrate marginal improvements in the power conversion efficiency (PCE) and fill factor (FF) for the devices fabricated with Turbo-Coat.

Additionally, there is a notable narrowing in the distribution range for all optoelectronic characteristics except for short-circuit current (J${}_{\text{sc}}$). This reduction is especially pronounced in the open circuit voltage (V${}_{\text{oc}}$) indicated by the reduction of the standard deviation from 0.2 for the commercial device to 0.05 for our device (Table 2).

**Fig. 15:**
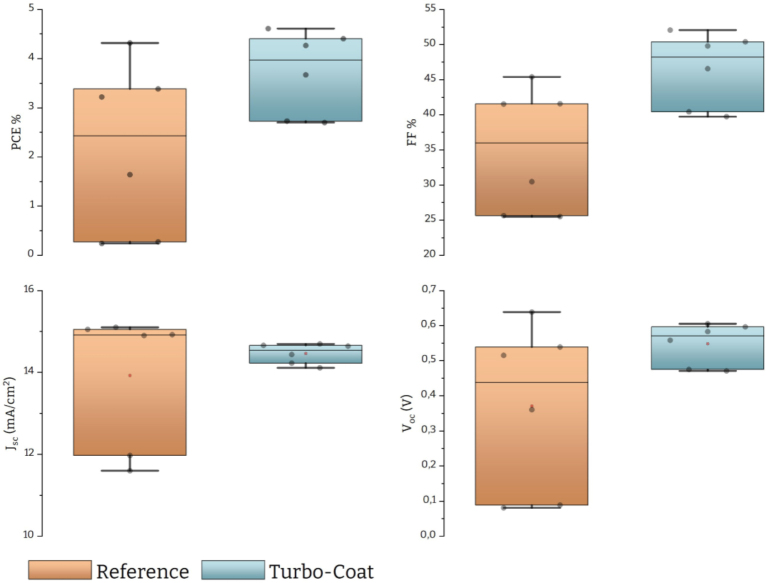
Optoelectronic characteristics of Sb${}_{\text{2}}$S${}_{\text{3}}$ champion solar cells fabricated with reference and Turbo-Coat spin coaters.

V${}_{\text{oc}}$ is a common indicator of the quality and uniformity of the semiconducting film and thus consistency across the devices shows uniform film formation and increased repeatability. A well-organized crystal structure is crucial for efficient charge transport, which can contribute to a higher V${}_{\text{oc}}$. Non-uniformities or variations in the thin film’s composition or thickness can lead to localized variations in the electric field and charge carrier concentrations. These variations can affect the voltage distribution within the cell, impacting the open-circuit voltage. Finally, to further investigate the effect of film uniformity we examined the same values for the champion devices of each batch Fig. 15. These data represent a single solar cell (i.e., a single semiconductor thin film) divided into 4 pixels suggesting that any deviations between values should be attributed to nonuniform film formation during spin coating. As expected the distributions are greatly reduced in the case of our device, indicating uniform isotropic film across all regions.

**Table 2:** Descriptive statistics of PCE and V${}_{\text{oc}}$ over 38 data points.

		N total	Mean	Standard Deviation	Sum	Minimum	Median	Champion Cells
%PCE	Reference	38	2.69	1.40	102.21	0.14	3.28	4.32
	Turbo-coat	38	3.14	0.74	119.37	1.56	3.26	4.61

V${}_{\text{oc}}$(V)	Reference	38	0.48	0.20	18.37	0.056	0.59	0.64
	Turbo-coat	38	0.58	0.05	22.19	0.45	0.60	0.64

In conclusion, the device presented in this work was found to be capable of producing high-quality semiconducting films for solar cell applications. Additionally, when compared to the commercial analogue, it produced more repeatable and potentially more uniform thin films, resulting in solar cells with higher performance and less deviation.

## CRediT authorship contribution statement

**Apostolos Kalafatis:** Writing – original draft, Visualization, Validation, Methodology, Investigation, Formal analysis, Data curation, Conceptualization. **Lazaros Theofylaktos:** Validation, Formal analysis, Data curation. **Thomas Stergiopoulos:** Writing – review & editing, Supervision, Funding acquisition.

## Declaration of competing interest

The authors declare the following financial interests/personal relationships which may be considered as potential competing interests: Thomas Stergiopoulos reports financial support was provided by European Research Council. If there are other authors, they declare that they have no known competing financial interests or personal relationships that could have appeared to influence the work reported in this paper.
